# Responsiveness of the PROMIS® measures to changes in disease status among pediatric nephrotic syndrome patients: a Midwest pediatric nephrology consortium study

**DOI:** 10.1186/s12955-017-0737-2

**Published:** 2017-08-23

**Authors:** David T. Selewski, Jonathan P. Troost, Danyelle Cummings, Susan F. Massengill, Rasheed A. Gbadegesin, Larry A. Greenbaum, Ibrahim F. Shatat, Yi Cai, Gaurav Kapur, Diane Hebert, Michael J. Somers, Howard Trachtman, Priya Pais, Michael E. Seifert, Jens Goebel, Christine B. Sethna, John D. Mahan, Heather E. Gross, Emily Herreshoff, Yang Liu, Noelle E. Carlozzi, Bryce B. Reeve, Darren A. DeWalt, Debbie S. Gipson

**Affiliations:** 1 Division of Nephrology, Department of Pediatrics & Communicable Diseases, C.S. Mott Children’s Hospital, University of Michigan, 1500 E Medical Center Drive, SPC5297, Ann Arbor, MI 48109-5297 USA; 2 Division of Pediatric Nephrology, Levine Children’s Hospital, Charlotte, NC USA; 3 Department of Pediatrics, Division of Nephrology, Duke University Medical Center, Durham, NC USA; 4 Department of Pediatrics, Emory University and Children’s Healthcare of Atlanta, Atlanta, GA USA; 5 Pediatric Nephrology and Hypertension, Sidra Medical and Research Center, Doha, Qatar; 6 DeVos Children’s Hospital, Grand Rapids, MI USA; 7 Pediatric Nephrology and Hypertension Division, Children’s Hospital of Michigan, Detroit, MI USA; 8 Division of Nephrology, The Hospital for Sick Children, Toronto, ON Canada; 9 Division of Nephrology, Boston Children’s Hospital, Harvard Medical School, Boston, MA USA; 10 Department of Pediatrics, Division of Nephrology, New York University Langone Medical Center, New York, NY USA; 11 Pediatric Nephrology and Hypertension, Medical College of Wisconsin, Milwaukee, WI USA; 12 Division of Pediatric Nephrology, Department of Pediatrics, University of Alabama Birmingham, Birmingham, AL USA; 13 Division of Nephrology and Hypertension, Cincinnati Children’s Hospital, Cincinnati, OH USA; 14 Division of Pediatric Nephrology, Cohen Children’s Medical Center of New York, New Hyde Park, NY USA; 15 Nationwide Children’s Hospital, The Ohio State University, College of Medicine, Columbus, OH USA; 16 University of North Carolina, Chapel Hill, NC USA; 17 Department of Psychology, University of North Carolina, Chapel Hill, NC USA; 18 Department of Physical Medicine and Rehabilitation, University of Michigan, Ann Arbor, USA

**Keywords:** Patient-reported outcomes, Quality of life, Nephrotic syndrome, Pediatrics, Children

## Abstract

**Background:**

Nephrotic syndrome represents a condition in pediatric nephrology typified by a relapsing and remitting course, proteinuria and the presence of edema. The PROMIS measures have previously been studied and validated in cross-sectional studies of children with nephrotic syndrome. This study was designed to longitudinally validate the PROMIS measures in pediatric nephrotic syndrome.

**Methods:**

One hundred twenty seven children with nephrotic syndrome between the ages of 8 and 17 years participated in this prospective cohort study. Patients completed a baseline assessment while their nephrotic syndrome was active, a follow-up assessment at the time of their first complete proteinuria remission or study month 3 if no remission occurred, and a final assessment at study month 12. Participants completed six PROMIS measures (Mobility, Fatigue, Pain Interference, Depressive Symptoms, Anxiety, and Peer Relationships), the PedsQL version 4.0, and two global assessment of change items.

**Results:**

Disease status was classified at each assessment: nephrotic syndrome active in 100% at baseline, 33% at month 3, and 46% at month 12. The PROMIS domains of Mobility, Fatigue, Pain Interference, Depressive Symptoms, and Anxiety each showed a significant overall improvement over time (*p* < 0.001). When the PROMIS measures were compared to the patients’ global assessment of change, the domains of Mobility, Fatigue, Pain Interference, and Anxiety consistently changed in an expected fashion. With the exception of Pain Interference, change in PROMIS domain scores did not correlate with changes in disease activity. PROMIS domain scores were moderately correlated with analogous PedsQL domain scores.

**Conclusion:**

This study demonstrates that the PROMIS Mobility, Fatigue, Pain Interference, and Anxiety domains are sensitive to self-reported changes in disease and overall health status over time in children with nephrotic syndrome. The lack of significant anchoring to clinically defined nephrotic syndrome disease active and remission status may highlight an opportunity to improve the measurement of HRQOL in children with nephrotic syndrome through the development of a nephrotic syndrome disease-specific HRQOL measure.

**Electronic supplementary material:**

The online version of this article (doi:10.1186/s12955-017-0737-2) contains supplementary material, which is available to authorized users.

## Background

Nephrotic syndrome is a challenging disease typified by a complex disease spectrum, including a single episode, relapsing and remitting disease, and persistent progressive disease resulting in end stage kidney disease in the worst cases [1]. Nephrotic syndrome can result in multiple complications, frequent hospitalizations, and upheaval of children’s day to day lives. Furthermore, nephrotic syndrome frequently can become a chronic disease that continues throughout the critical years of a child’s development and persists into adulthood. Although we have made significant improvements in clinical care, these children continue to experience difficulties in functioning. In order to better understand these functional limitations, longitudinal assessments of health-related quality of life (HRQOL) are imperative.

HRQOL is impacted in nephrotic syndrome by symptoms related to the disease (edema), medications (e.g., corticosteroids, calcineurin inhibitors), and the unpredictable relapsing-remitting nature of the disease [2, 3]. Although cross-sectional evaluations have shown that NS adversely impacts HRQOL in physical, social, and emotional domains [2–4], there is an absence of published data about the longitudinal trends in HRQOL in this population. The NIH Sponsored Patient-Reported Outcomes Measurement Information System® (PROMIS®) has expanded our ability to measure and describe the HRQOL in children with chronic disease by creating measures that are efficient, precise, and valid across a variety of diseases to assess patient-reported outcomes (PROs) (www.HealthMeasures.net) [5–9]. Pediatric nephrotic syndrome has been incorporated as one of the exemplar chronic conditions in the initial PROMIS I and PROMIS II validation studies [10]. The initial PROMIS validation work in pediatric nephrotic syndrome showed the measures were associated with disease status. Specifically, children with active nephrotic syndrome (i.e., presence of edema) had more Fatigue, Pain Interference, and Anxiety and less Mobility than children with inactive disease [3]. In addition, work has shown that children with prevalent disease experience more Pain Interference and are less satisfied with their Peer Relationships than children with incident disease [11]. Furthermore, our previous work has shown that the PROMIS measures provide complimentary results to a legacy measure, PedsQL™ 4.0 Generic Scales (PedsQL), with important differences that likely reflect differences in measure content [12].

A critical next step in the validation process is a longitudinal assessment of the ability of the PROMIS measures to detect change over time. In order to address this need, this study was designed to: (1) comprehensively describe the longitudinal HRQOL experience in pediatric nephrotic syndrome, (2) evaluate the responsiveness of PROMIS measures to self-reported global assessments of change, (3) evaluate the responsiveness of PROMIS to changes in disease state, and to (4) assess the consistency of the findings of the PROMIS and a legacy instrument (PedsQL).

## Methods

### Study design and participants

The PROMIS II nephrotic syndrome longitudinal cohort study enrolled children with active nephrotic syndrome from 14 participating centers (12 from the Midwest Pediatric Nephrology Consortium (United States and Canada) and 2 additional centers in the United States). Each site obtained institutional IRB approval. Parents and children gave informed consent and assent respectively prior to enrollment in this study.

The PROMIS II nephrotic syndrome cohort included children 8–17 years. Children with active nephrotic syndrome defined as the presence of nephrotic range proteinuria (>2+ urinalysis and edema or urine protein/creatinine ratio (UPC) >2 g/g) were eligible. Exclusion criteria included co-existing medical, psychiatric, or cognitive impairments that would prevent the patient from answering the computer administered questionnaire or the inability to speak and read English.

The longitudinal study design included three study visits. Patients completed a baseline assessment while their nephrotic syndrome was active. Patients completed an event assessment (event visit) when: 1) they reached complete remission or 2) three months after their baseline visit if remission did not occur. This event visit was conducted on-line. Finally, all patients completed a third visit 12 months after their baseline visit. While all patients were active at baseline, patients could either have active disease or be in remission at their two follow-up visits. Remission was defined as normalization of urinary protein excretion and complete resolution of edema. Data on symptom burden, past medical history and other health conditions, demographic information, medication, and health care utilization were collected at each visit. A physical evaluation measuring edema, blood pressure, and body size measurements was done at baseline and at month 12. Laboratory data, such as urine protein: creatinine ratio (UPC) and eGFR, were also collected at baseline and month 12, but not at the event visit.

### Collection of PRO data

The PROMIS Pediatric measures included PROMIS measures of physical health (Mobility, Fatigue, Pain Interference), mental health (Depressive Symptoms and Anxiety) and social health (Peer Relationships). We used the PROMIS Assessment Center web-based interface (www.assessmentcenter.net) to administer computer-adaptive testing (CAT) questionnaires. Each PROMIS question used the context statement “In the past 7 days.” Responses included 5 options ranging from “never” to “almost always” in the majority of domains and from “with no trouble” to “not able to do” for the Mobility measure. Each PROMIS Pediatric measure generates a T-score (mean = 50, standard deviation (SD) = 10 in the calibration population) [6]. Higher scores indicate higher levels of the trait that is being measured (i.e., higher scores signify worse Depression, Anxiety, Fatigue, and Pain Interference, and better functioning for Mobility and Peer Relationships). Administration time for all PROMIS measures using CAT was approximately six minutes.

The PedsQL is a legacy instrument designed to measure HRQOL in children. The instrument measures physical, emotional, social, school, and overall functioning. This instrument has been evaluated in healthy children and in multiple pediatric chronic conditions including chronic kidney disease [13]. The PedsQL asks participants to review statements and rate the degree that the subject has experienced that symptom in the past week. The domain scores result from the summation of problem frequency within each domain. Scores range from 0 to 100 with higher scores indicating better function. Mean scores in the general population range from 78 to 87 depending on the domain (standard deviations range from 13 to 19 [12]. Administration time was approximately ten minutes.

In addition to standardized assessments of PROs, patients were also asked questions to determine their global assessment of change for nephrotic syndrome and for overall health. At the event (post baseline assessment) and month 12 visits, patients were asked “How is your nephrotic syndrome compared to your last study visit?,” and “How is your overall health compared to your last study visit?” each with possible responses of “Better,” “About the same,” and “Worse.”

Patient clinical classification was assigned at each study visit as nephrotic syndrome disease remission defined as normalization of urinary protein excretion and complete resolution of edema. Patients who were not in a remission were considered to have active disease.

### Statistical analysis

Descriptive statistics on key variables of interest were provided as frequencies and percentages with means and SD as appropriate. Changes in scores across follow-up visits were tested using a repeated measures ANOVA analysis. Statistical significance was tested using *F*-tests, and effect sizes were measured using an eta-squared.

Changes in scores for all PRO measures were calculated by subtracting the patient’s score from their baseline assessment. These values were then compared across response categories to the global assessment of change items with the hypothesis that those reporting improvements in their nephrotic syndrome or general health would be more likely to have improvements in PRO scores. Statistical significance for these comparisons was assessed using a repeated measure ANOVA. Analogous repeated measures ANOVAs were conducted to test for a difference in PRO scores over time by proteinuric remission status at follow-up. We also tested if global assessment of change or remission status was associated with a significant change greater than the published Pediatric PROMIS domain score minimally important difference of 3 [14]. To test this, we examined if the 95% confidence interval of the mean change for each group contained the minimally important difference value of 3 [14].

Finally, changes in PROMIS scores were compared to changes in PedsQL scores using Pearson correlation coefficients.

## Results

There were 127 patients who completed a baseline visit, 112 with a completed event visit and 90 with a 12-month follow-up visit. The cohort was relatively equally distributed by age (8–12 vs. 13–17 years old) and disease duration (Incident vs. Prevalent). The median days from baseline to first follow-up in those reaching remission was 39 (IQR 20, 81) compared to 104 (IQR 89, 120) in those not reaching remission (*p* < 0.0001). A total of 75 of 112 participants were in remission at the event visit and 49 of 90 participates were in remission at the 12-month visit. Patient demographics and clinical characteristics of the sample are presented in Table 1. The cohort at baseline was notable for edema in 76%, immunosuppression in 78%, and hospitalized within the last 6 months in 46%.

**Table 1:** PROMIS II patient characteristics at baseline

	N(%), or Mean(std)
Disease duration	
Incident, n(%)	60 (47)
Prevalent, n(%)	67 (53)
Age	
8–12 years, n(%)	67 (53)
13–17 years, n(%)	60 (47)
Female, n(%)	44 (35)
Race	
White, n(%)	65 (52)
Black, n(%)	36 (28)
Asian, n(%)	16 (13)
Other, n(%)	10 (8)
Hispanic or Latino ethnicity, n(%)	10 (8)
Maternal Education	
< High School, n(%)	12 (9)
High school, n(%)	46 (36)
2-Year Associate’s Degree, n(%)	16 (13)
4-Year College Degree, n(%)	24 (19)
Graduate Degree, n(%)	7 (6)
Unknown, n(%)	22 (17)
Obese, n(%)	44 (35)
Edema Present, n(%)	96 (76)
Hypertension, n(%)	53 (42)
Number of Symptoms, Mean(std)	4 (3)
Number of Medical Conditions, Mean(std)	1 (1)
Immunosuppressive therapy, n(%)	99 (78)
Emergency Department Visits in last 6 months (≥ 1 visit), n(%)	49 (39)
Hospitalizations in last 6 months (≥ 1 visit), n(%)	58 (46)
Estimated GFR (mL/min/1.73 m^2^), Mean(std)	113 (48)
Urine Protein:Creatinine, Mean(std)	6.7 (7.1)
Albumin, Mean(std)	2.4 (1.0)
Hemoglobin, Mean(std)	13.3 (2.0)
*Number of medications, Mean(std)*	*3 (3)*

Table 2 shows the mean scores for the PROMIS and PedsQL domains at each visit. Over time the HRQOL scores improved in each of the domains in both instruments. On average, patients had more favorable HRQOL scores (e.g., more Mobility; less Fatigue) during follow-up than at baseline. For example, the baseline mean Mobility score of 46.3 increased to 52.4 at month 12. All of the PROMIS and PedsQL domains tested changed significantly over time with the exception of PROMIS Peer Relationships and PedsQL Social Functioning. The same pattern was observed when limiting the analyses to 85 patients with PROMIS assessments at all three study visits. A separate analysis was performed and noted that change in PROMIS and PedsQL scores did not differ significantly by race at the event visit or the 12-month visit.

**Table 2:** PROMIS II HRQOL domain scores at each assessment

	Baseline		Event visit		Month 12				
	*N* = 127		*N* = 112		*N* = 90				
PROMIS	Mean(SD)	Clinically impaired N(%)*	Mean(SD)	Clinically impaired N(%)*	Mean(SD)	Clinically impaired N(%)*	F-value	*p*-value	η^2^
Mobility	46.3 (9.2)	31 (25)	50.8 (9.5)	14 (13)	52.4 (9.2)	9 (10)	12.81	<0.001	0.074
Fatigue	49.6 (12.6)	29 (23)	43.4 (12.7)	9 (8)	43.3 (13.4)	11 (12)	9.16	<0.001	0.054
Pain Interference	49.7 (11.1)	24 (20)	44.9 (11.2)	7 (6)	44.7 (10.9)	10 (11)	7.44	<0.001	0.045
Depression	49.7 (9.3)	22 (18)	45.2 (9.9)	8 (7)	46.2 (9.6)	6 (7)	7.04	0.001	0.042
Anxiety	49.4 (10.6)	23 (19)	43.2 (11.5)	11 (10)	44.5 (10.7)	7 (8)	10.64	<0.001	0.062
Peer Relationships	48.6 (10.6)	23 (19)	50.4 (11.3)	16 (15)	51.7 (10.4)	7 (8)	2.15	0.12	0.013
PedsQL									
Physical functioning	69.3 (22.7)	59 (47)	79.1 (20.8)	29 (27)	81.5 (21.1)	25 (28)	10.04	<0.001	0.059
Emotional functioning	72.3 (22.1)	61 (48)	80.1 (20.9)	33 (30)	80.5 (20.9)	27 (30)	5.42	0.005	0.032
Social functioning	80.6 (18.0)	25 (20)	84.0 (18.2)	18 (17)	85.2 (18.5)	16 (18)	1.91	0.15	0.012
School functioning	63.7 (21.2)	38 (35)	76.9 (20.3)	15 (16)	74.7 (21.1)	20 (25)	11.57	<0.001	0.076
Overall	71.0 (17.0)	44 (35)	80.0 (17.1)	21 (19)	80.5 (18.2)	20 (22)	10.91	<0.001	0.064

*>1SD from the mean in the clinically impaired direction. For PROMIS Mobility, and Peer Relationships, a higher score indicates better patient reported outcomes; for PROMIS Fatigue, Pain Interference, Depression, and Anxiety a higher score indicates worse patient reported outcomes. For all PedsQL domains, a higher score indicates better patient reported outcomes

**Fig. 1:**
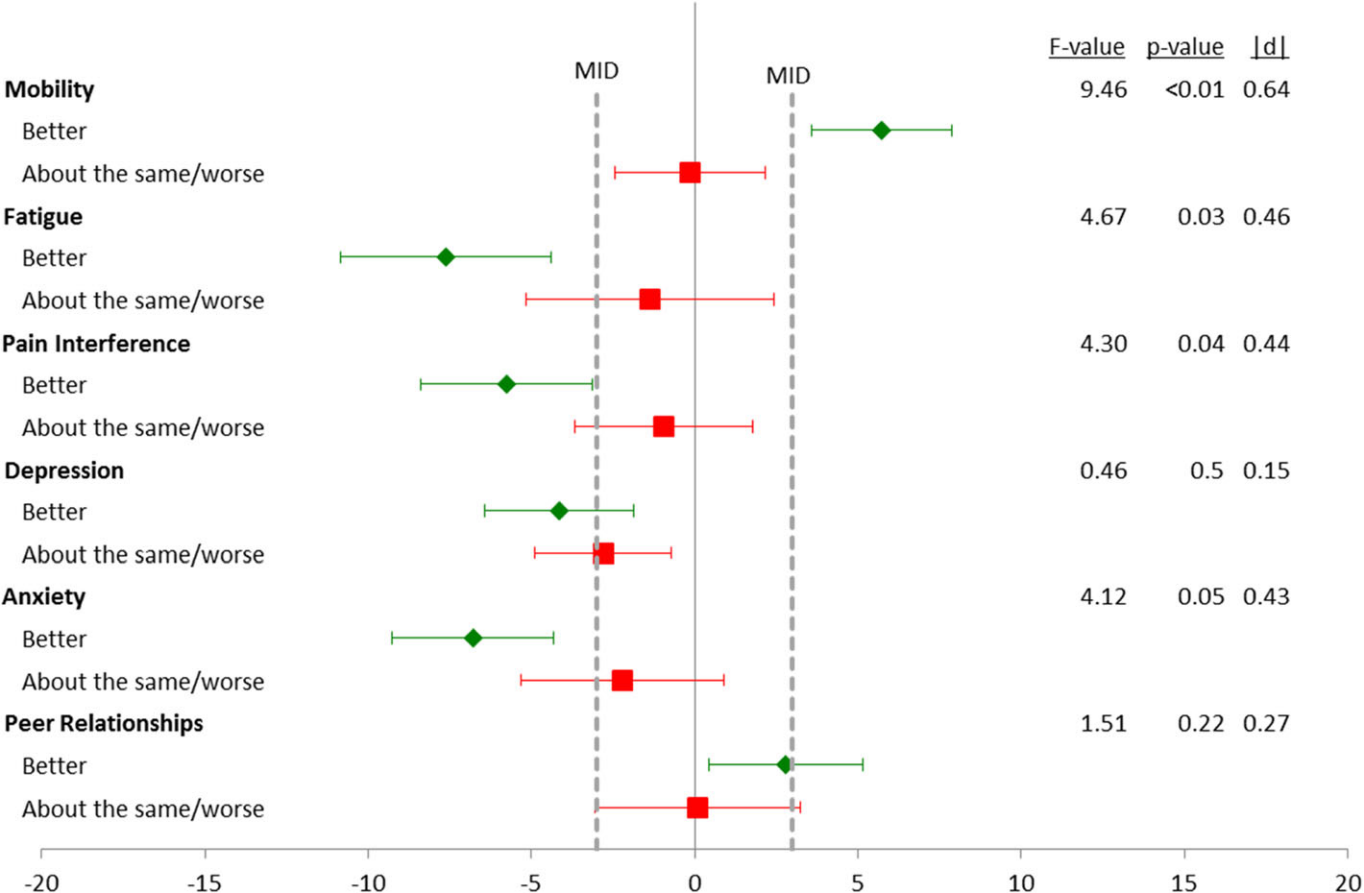
Change in PROMIS scores from baseline to event visit by self-reported Global Assessment of Change in nephrotic syndrome since last study visit. Results are shown as mean change with 95% confidence intervals. Group Sample sizes: Better = 78, About the same = 25, Worse = 5. Minimally Important Difference (MID)

When the PROMIS scores were compared to the patients’ global assessment of change in nephrotic syndrome at the event visit, PROMIS Mobility, Fatigue, Pain Interference, and Anxiety changed in an expected fashion: patients reporting an improvement in their nephrotic syndrome showed an improvement in their PRO (Fig. 1). For example, the 78 participants who reported that their nephrotic syndrome had improved since their last visit had a mean increase in Mobility of 5.8 points. The 30 participants who indicated their nephrotic syndrome was “about the same” or “worse” on average had a mean decrease in Mobility of 0.1 points (|d| = 0.64). The MID of |3| is indicated on Fig. 1 with a dotted line. Patients reporting that their nephrotic syndrome was “better” had an improvement in Mobility, Fatigue, Pain Interference, and Anxiety at a magnitude significantly greater than the MID of 3 based on 95% confidence intervals. For example, as stated above, patients reporting that their NS was “better” had a mean increase in mobility of 5.8 points, and the 95% confidence interval for this estimate ranged from 3.6 to 7.9 indicating a statistically significant change of more than 3.0 (one-sample t-test *p* = 0.007).

**Fig. 2:**
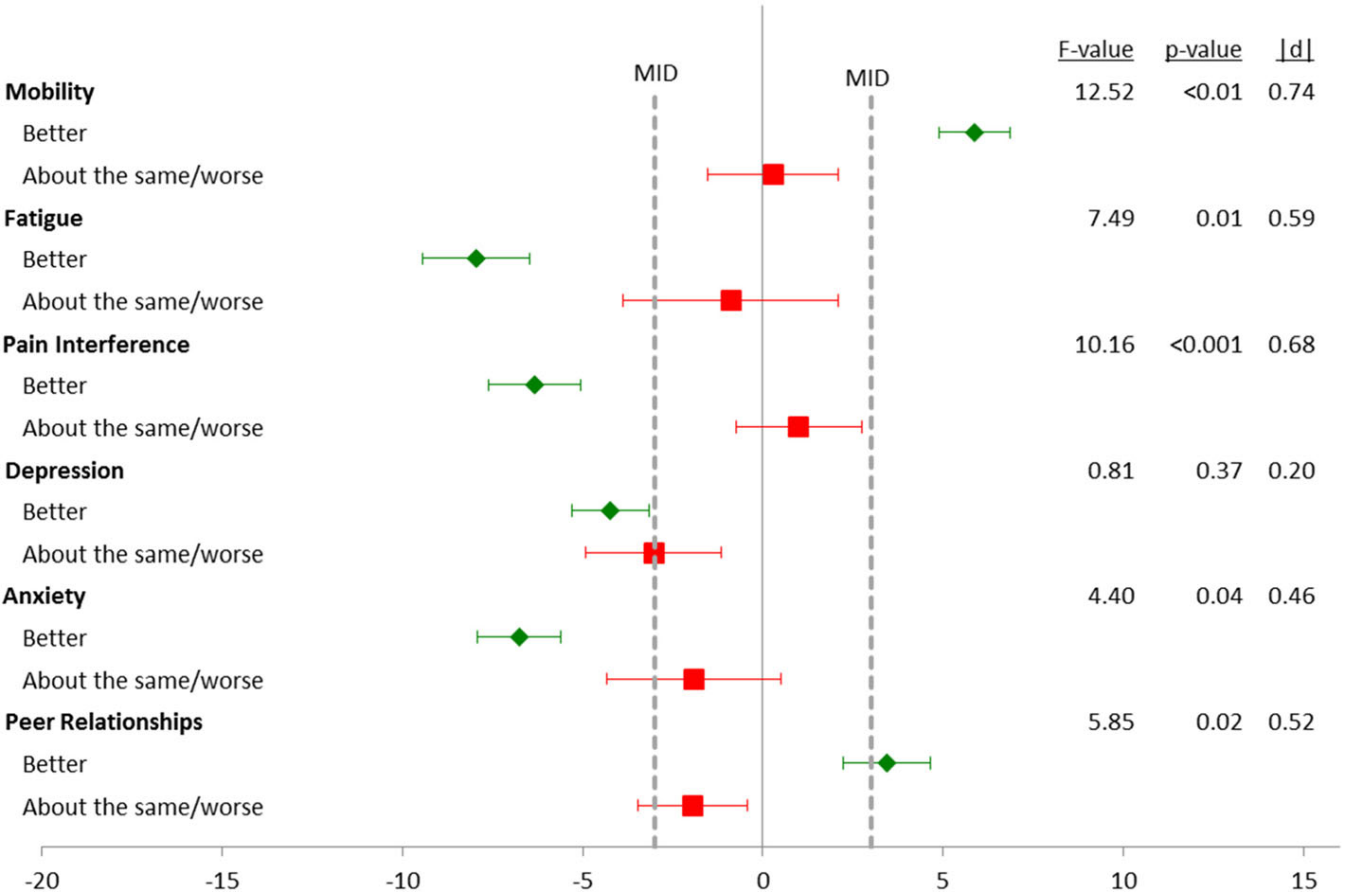
Change in PROMIS scores from baseline to event visit by Global Assessment of Change in overall health since last study visit. Results are shown as mean change with 95% confidence intervals. Sample sizes: Better = 80, About the same = 22, Worse = 6. Minimally Important Difference (MID)

Figure 2 contains similar estimates comparing change in PRO to the patient’s reported change in overall health. As with the global assessment of nephrotic syndrome, there were significant differences by global assessment of overall health and changes of PROMIS Mobility, Fatigue, Pain Interference, and Anxiety. There was also a significant difference in Peer Relationships (|d| = 0.52, *p* = 0.02), however, the degree of change in Peer Relationships for the “better” group was not greater than the MID of 3.

**Fig. 3:**
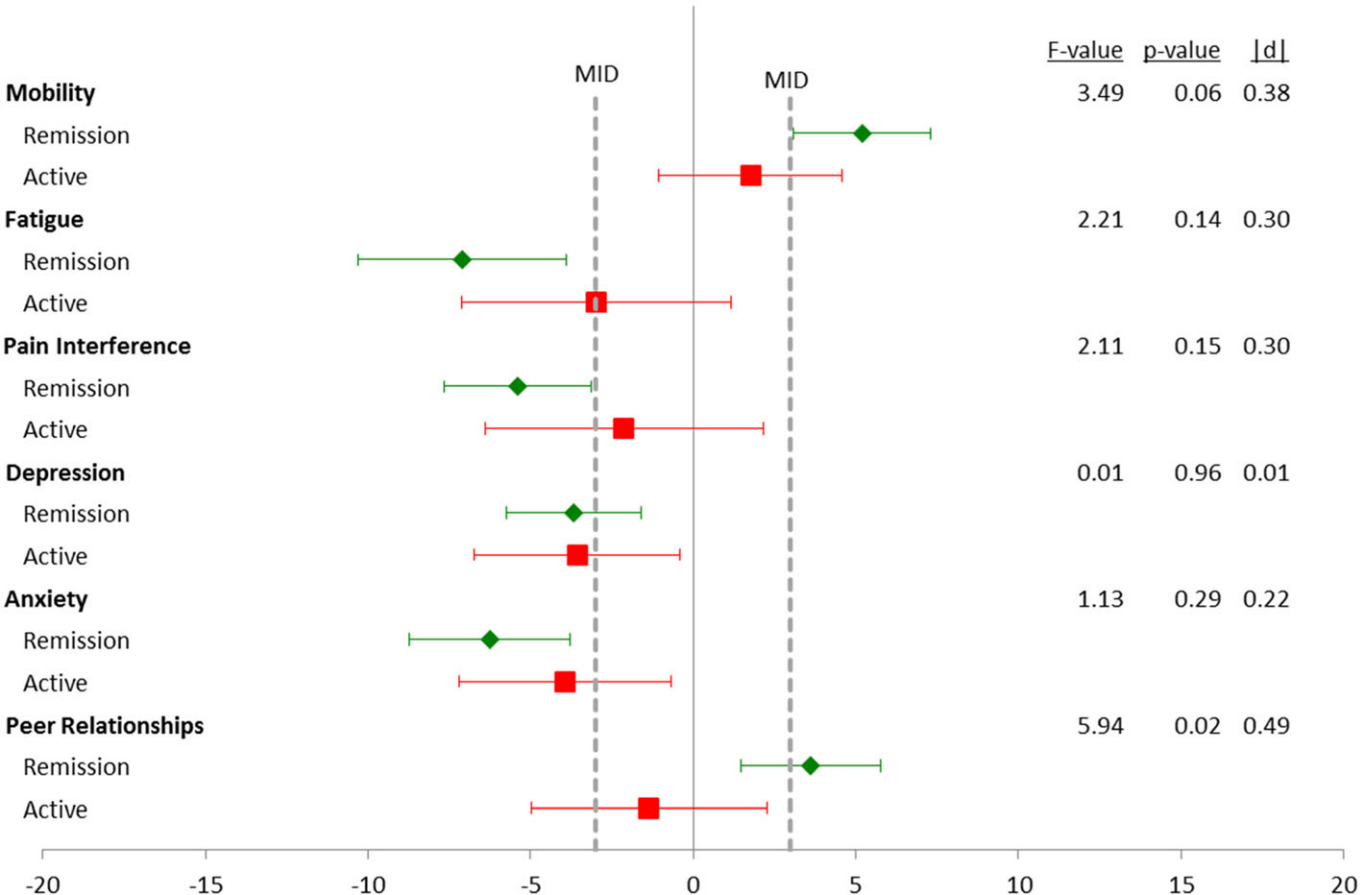
Change in PROMIS scores from baseline to event visit by clinical disease status based on proteinuria remission status at event visit. All participants were disease active at baseline. Remission was defied as normalization of urinary protein excretion and complete resolution of edema. Results are shown as mean change with 95% confidence intervals. Sample sizes: Remission = 75, Active = 37. Minimally Important Difference (MID)

Figure 3 compares changes in PRO by changes in clinical disease activity during follow-up. As part of the study design all patients had active disease at baseline, but disease activity varied during follow-up visits. While there was a significant relationship between proteinuria remission at the event visit and mean change in Peer Relationships score (*p* = 0.02 |d| = 0.49), no other domains had a statistically significant relationship between change in PRO and disease activity at the event visit. Additionally, those in the remission group did, on average, improve their Peer Relationships scores by more than the MID of 3.0.

**Fig. 4:**
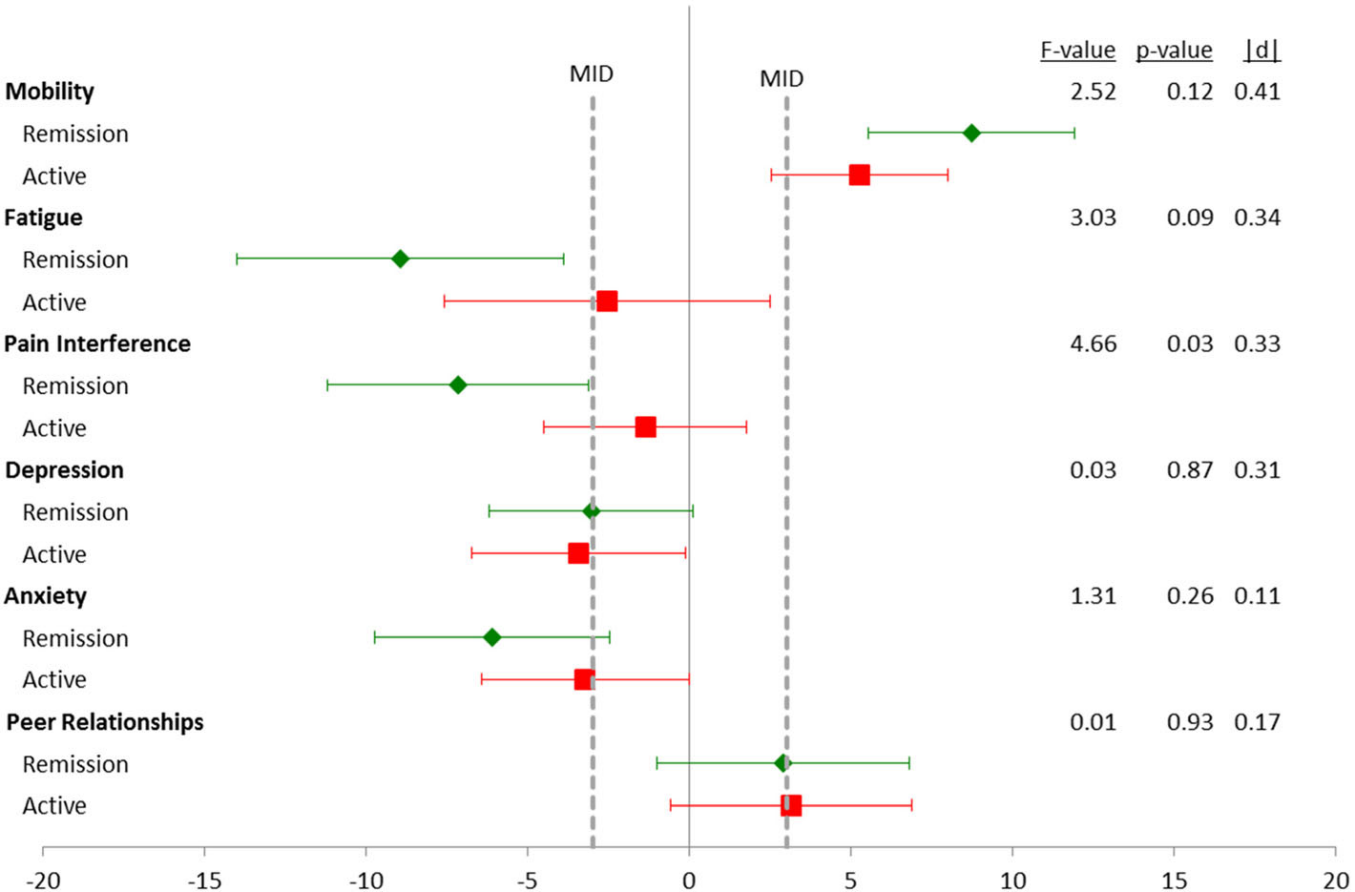
Change in PROMIS scores from baseline to month 12 visit. Participants were classified by proteinuria remission status at month 12. All participants were disease active at baseline. Remission was defied as normalization of urinary protein excretion and complete resolution of edema. Results are shown as mean change with 95% confidence intervals. Sample sizes: Remission = 49, Active = 41. Minimally Important Difference (MID)

Figure 4 compares changes in PRO by remission status at month 12. There was a significant difference in change of Pain Interference scores by remission status where those achieving a remission had a mean decrease in Pain Interference of 7.2 points from their active baseline visit, and those with persistently active disease had a mean decrease of 1.4 (|d| = 0.33, 0.03). Apart from this difference in Pain Interference scores, there was no significant difference in change in PROMIS scores by remission status at month 12.

Finally, correlations of the changes in PROMIS scores with the changes in PedsQL scores are displayed in Table 3. Similar domains had stronger correlations. For example, PROMIS Mobility and PedsQL Physical Functioning had a correlation of *r* = 0.60; PROMIS Anxiety and PedsQL Anxiety had a correlation of *r* = −0.68 (i.e., higher PROMIS Anxiety correlated with worse PedsQL emotional functioning, as expected). PROMIS Peer Relationships was not correlated with PedsQL Social Functioning (*r* = 0.11). Analogous analyses of the responsiveness of the PedsQL to changes in global assessment of change and to remission status are found in Additional file 1 Fig. S1, Additional file 2: Fig. S2, Additional file 3: Fig. S3 and Additional file 4: Fig. S4. The PedsQL Physical Functioning domain showed the strongest relationship to global assessment of change in nephrotic syndrome and overall health (|d| = 0.65 and 0.72 respectively). Remission status at the event visit was not associated with changes in any of the PedsQL domains. Remission status at the month 12 visit was associated with greater improvement in PedsQL Physical, Emotional, and Social functioning.

**Table 3:** Correlation between changes in PRO domains

	Peds QL Domains				
PROMIS Domain	Physical Functioning	Emotional Functioning	Social Functioning	School Functioning	Overall HRQOL
Physical Functioning					
Mobility	0.60	0.39	0.45	0.37	0.60
Fatigue	−0.65	−0.57	−0.48	−0.59	−0.73
Pain Interference	−0.63	−0.52	−0.44	−0.46	−0.66
Emotional Functioning					
Depressive Symptoms	−0.46	−0.55	−0.44	−0.39	−0.57
Anxiety	−0.51	−0.68	−0.35	−0.51	−0.64
Social Functioning					
Peer Relationships	0.13	0.18	0.11	0.10	0.16

Estimates shown are Pearson correlation coefficients

## Discussion

This comprehensive longitudinal study evaluated PROMIS pediatric domains in children with nephrotic syndrome for the purposes of validation. The PROMIS scores in this disease group were assessed longitudinally and compared to three types of anchor measurements (1) self-reported global assessment of change, (2) a clinical marker of disease status, and (3) a legacy HRQOL instrument. We demonstrated that the Mobility, Fatigue, Pain Interference, and Anxiety domains were significantly associated with both the self-reported global assessments of change, and the legacy HRQOL instrument. However, we did not find that these domains were associated with a clinical marker of disease status. Overall, we recommend this subset of PROMIS domains for use in measuring HRQOL over time in pediatric patients with nephrotic syndrome.

As the care of children with chronic illnesses has improved there has been a shift toward improving their global care by fostering understanding of the impact of disease on HRQOL. The PROMIS Pediatric measures assess HRQOL in children between 8 and 17 years of age. The PROMIS Pediatric measures have been validated in a number of childhood chronic illnesses including cancer, asthma, obesity, chronic kidney disease, and NS in the PROMIS I cross-sectional study [3, 15–17]. The cross-sectional study of children with NS demonstrated that the PROMIS Pediatric domains of Mobility, Pain Interference, Depressive Symptoms, Anxiety, and Peer Relationships were associated with the clinical status of children with chronic kidney disease [16]. In an analysis of the 151 children with nephrotic syndrome in the PROMIS I study, children with active NS, defined by presence of edema, had significantly worse scores in the domains of Mobility, Fatigue, Pain Interference, and Anxiety when compared to children with inactive nephrotic syndrome [3]. The results of this study add to this growing literature and illustrate that nephrotic syndrome appears to have a greater impact on physical health domains (i.e., Mobility, Fatigue, Pain Interference) than on mental or social domains.

These initial studies began to establish the feasibility and validity of the PROMIS measures in children with nephrotic syndrome. The current study takes a critical step in extending the assessment of PROMIS measure validation by assessing the responsiveness of the instrument to change in disease status over time. The current study demonstrates that PROMIS measures are capable of detecting significant changes over time that correlate with global changes in the patients’ self-reported disease status. In evaluating the clinical utility of any measure, it is imperative to be able to interpret a clinically meaningful change in a measured value, and to do this clinicians and researchers may employ the concept of minimally important difference in HRQOL measures. A minimally important difference is defined as the “smallest difference in score … that patients perceive as important, … and which would lead the clinician to consider a change in the patient’s management” [14, 18–20]. A minimally important difference of 3 points in PROMIS Pediatric measures has been defined for children with nephrotic syndrome and other chronic health conditions through research including patients, parent caregivers, and physicians [14]. Based on this interpretation, the PROMIS Mobility, Fatigue, Pain Interference, and Anxiety measures were successful at detecting a greater than a 3-point change related to the patients’ global assessment of their nephrotic syndrome and of their overall health. We noted slightly stronger effect sizes for the changes in global health than for the changes in nephrotic syndrome. This difference is not surprising as the PROMIS domains are not disease specific, and may be more responsive to overall changes in health than to disease specific changes.

A telling feature of these analyses is that, with few exceptions, the PROMIS domain score changes did not distinguish between patients who achieved a proteinuric remission and those with continued active disease on follow up. At first glance, this may give clinicians pause when assessing the utility of the instrument. One possible explanation for these findings is that generic PRO domains may not adequately describe the nephrotic syndrome experience, and that perhaps a nephrotic syndrome disease-specific domain, that uses terms and content specific to nephrotic syndrome, may be needed. Jolly et al., have recently published a novel disease-specific instrument tailored to measure HRQOL in patients with Systemic Lupus Erythematosus. Their validation analyses revealed that that a greater amount of variance in the disease specific HRQOL instrument was explained by markers of disease activity and damage than was found for the generic SF-36 instrument [21]. However, it is also important to consider that a patient’s disease experience is defined by more than their clinical markers of disease activity. Even though changes in PROMIS domains did not correlate with changes in disease activity, we believe there is still value to measuring a patient’s HRQOL in addition to their remission status as these patient-assigned and clinician-assigned measures may provide complementary information. In aggregate with our other analyses, we believe this study provides evidence that these PROMIS domains are useful tools in studying HRQOL in pediatric nephrotic syndrome patients.

As part of the validation of any new instrument such as PROMIS, it is traditional to compare it to a validated legacy measure (PedsQL) in a longitudinal population. There is no validated HRQOL measure for children with nephrotic syndrome. The PedsQL was selected as a legacy measure for this study as it has been used in general and disease focused research. We previously have shown that the PROMIS measures and PedsQL provide similar results in a number of domains with the strongest correlations existing between the PedsQL emotional functioning domain and the PROMIS domains of Depressive Symptoms and Anxiety in a cross-sectional analysis of this cohort [11]. In contrast the weakest correlation was between the social function and school functioning domains in the PedsQL and the PROMIS Peer Relationships domain. The current study confirms these findings in a longitudinal analysis. Our longitudinal analyses revealed significant and moderately strong correlations in PROMIS Mobility, Fatigue, and Pain Interference with the PedsQL Physical Functioning, as well as a significant relationship between PROMIS Anxiety and PedsQL Emotional Functioning. The longitudinal analyses also identified no correlation between PROMIS Peer Relationships and PedsQL Social Functioning. This can be partially explained by the domains assessing separate social constructs. The PROMIS Peer Relationship items tend to reflect the quality of relationships (e.g., “My friends and I helped each other out”) while the PedsQL Social items test for problems getting along with others (e.g., “Other kids do not want to be my friend”). We show that the PROMIS and PedsQL provide complementary HRQOL information, but their imperfect correlations indicate that there remain important differences in the domains assessed by the instruments.

Although this study provides critical data about the responsiveness of the PROMIS measures in nephrotic syndrome, it is also important to acknowledge several study limitations. The PROMIS Pediatric measures are limited to ages 8–17 years. At the time of this study, only an English version and school age self-report version of the PROMIS Pediatric measures were available. Consequently, the results of this study will be most applicable to English language, school age children. Finally, the study utilized the available PROMIS measure domains. Since this study implementation there have been a number of new PROMIS domains developed that may also capture important aspects of the nephrotic syndrome disease experience. Future studies will benefit from patient participation in domain selection. Finally, it is important to consider that none of the three types of anchors we used for validation, (1) self-reported global assessment of change, (2) a clinical marker of disease status, and (3) a legacy HRQOL instrument, are validated, gold-standard measures of HRQOL.

## Conclusion

Patient-reported HRQOL measures are designed to gather the patient perspective on their own health and functional status. This study demonstrates that the PROMIS Mobility, Fatigue, Pain Interference, and Anxiety domains are sensitive to self-reported changes in disease and overall health status over time in children with nephrotic syndrome. However, the PROMIS measure did not distinguish between groups of patients who improved to clinical disease remission and patients who remained with active disease. HRQOLs measure information that is distinct from clinical laboratory assessments and thus should be expected to provide information that is complimentary to laboratory assessments. The lack of significant anchoring to clinically defined nephrotic syndrome disease active and remission status may highlight an opportunity to improve the measurement of HRQOL in children with nephrotic syndrome through the development of an additional nephrotic syndrome disease-specific HRQOL measure.

## Additional files

Additional file 1: Change in PedsQL scores from baseline to event visit by self-reported Global Assessment of Change in nephrotic syndrome since last study visit. (DOCX 37 kb)

Additional file 2: Change in PedsQL scores from baseline to event visit by Global Assessment of Change in overall health since last study visit. (DOCX 37 kb)

Additional file 3: Change in PedsQL scores from baseline to event visit by clinical disease status based on proteinuria remission status at event visit. (DOCX 34 kb)

Additional file 4: Change in PedsQL scores from baseline to month 12 visit. Participants were classified by proteinuria remission status at month 12. (DOCX 35 kb)

## Abbreviations

CAT: Computer-adaptive testing
HRQOL: Health-related quality of life
PedsQL: PedsQL™ 4.0 Generic Scales
PROMIS®: Patient-Reported Outcomes Measurement Information System®
PROs: Patient-reported outcomes
SD: Standard deviation
UPC: Urine protein/creatinine ratio.
