# Supplementary material for: Responsiveness of the PROMIS® measures to changes in disease status among pediatric nephrotic syndrome patients: a Midwest pediatric nephrology consortium study
Source: Health Qual Life Outcomes. 2017 Aug 23;15:166. doi: 10.1186/s12955-017-0737-2 (PMC5569504; doi:10.1186/s12955-017-0737-2)
Supplement: Supplementary file 2 — Change in PedsQL scores from baseline to event visit by Global Assessment of Change in overall health since last study visit. (DOCX 37 kb) [file 12955_2017_737_MOESM2_ESM.docx]

**Appendix 2. Change in PedsQL scores from baseline to event visit by Global Assessment of Change in overall health since last study visit.**


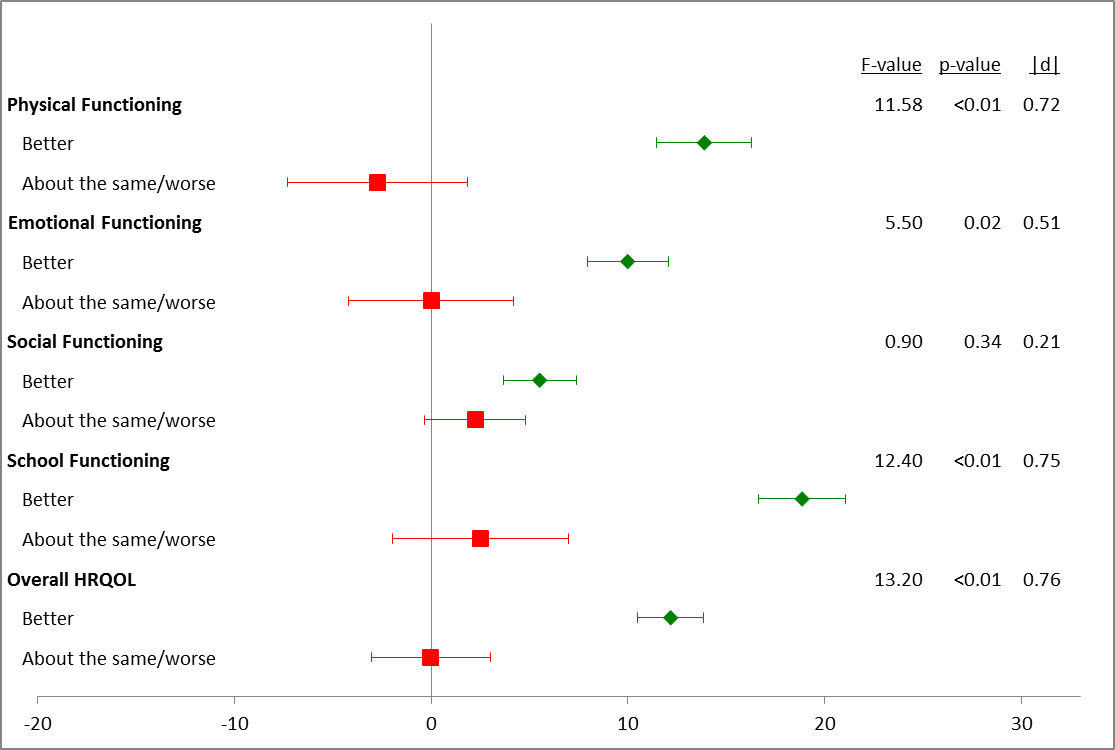


Results are shown as mean change with 95% confidence intervals. Sample sizes: Better=80, About the same=22, Worse=6
