# Supplementary material for: Responsiveness of the PROMIS® measures to changes in disease status among pediatric nephrotic syndrome patients: a Midwest pediatric nephrology consortium study
Source: Health Qual Life Outcomes. 2017 Aug 23;15:166. doi: 10.1186/s12955-017-0737-2 (PMC5569504; doi:10.1186/s12955-017-0737-2)
Supplement: Supplementary file 4 — Change in PedsQL scores from baseline to month 12 visit. Participants were classified by proteinuria remission status at month 12. (DOCX 35 kb) [file 12955_2017_737_MOESM4_ESM.docx]

**Appendix 4. Change in PedsQL scores from baseline to month 12 visit. Participants were classified by proteinuria remission status at month 12.**


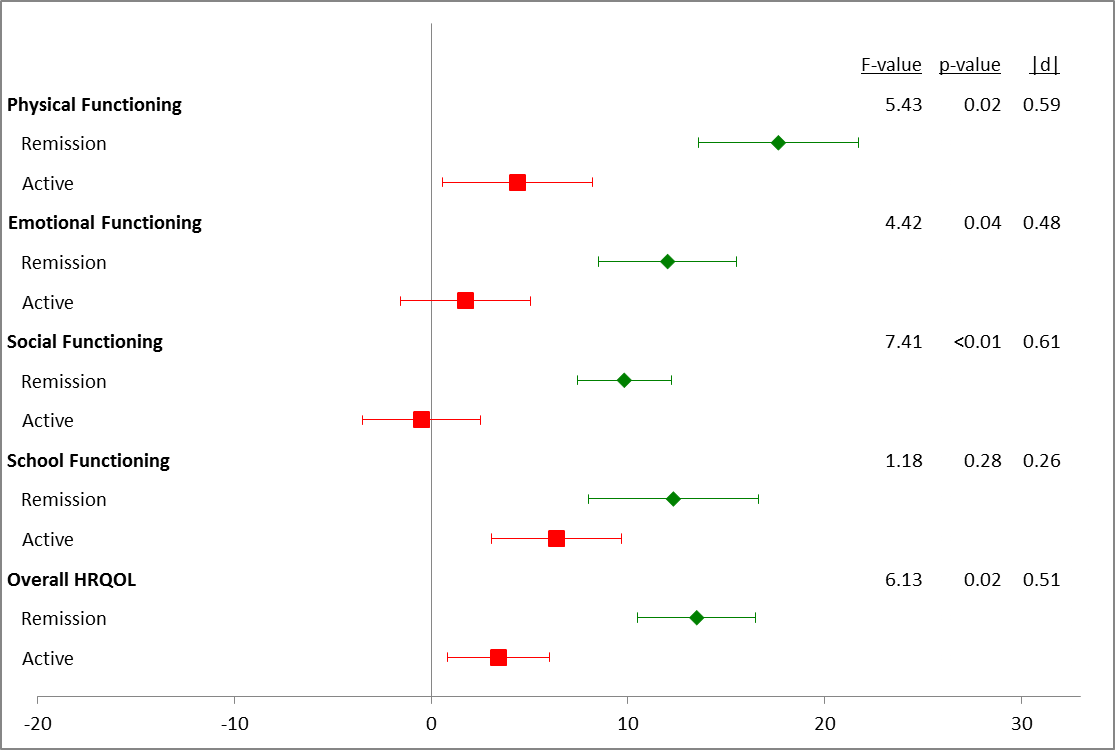


All participants were disease active at baseline. Remission was defied as normalization of urinary protein excretion and complete resolution of edema. Results are shown as mean change with 95% confidence intervals. Sample sizes: Remission=49, Active=41
